# Evolution and Functional Insights of Different Ancestral Orthologous Clades of Chitin Synthase Genes in the Fungal Tree of Life

**DOI:** 10.3389/fpls.2016.00037

**Published:** 2016-02-01

**Authors:** Mu Li, Cong Jiang, Qinhu Wang, Zhongtao Zhao, Qiaojun Jin, Jin-Rong Xu, Huiquan Liu

**Affiliations:** ^1^State Key Laboratory of Crop Stress Biology for Arid Areas, College of Plant Protection, Northwest A&F UniversityYangling, China; ^2^South China Botanical Garden, Chinese Academy of SciencesGuangzhou, China; ^3^Department of Botany and Plant Pathology, Purdue UniversityWest Lafayette, IN, USA

**Keywords:** chitin synthase, fungi, evolution, functional diversification, plant infection

## Abstract

Chitin synthases (CHSs) are key enzymes in the biosynthesis of chitin, an important structural component of fungal cell walls that can trigger innate immune responses in host plants and animals. Members of CHS gene family perform various functions in fungal cellular processes. Previous studies focused primarily on classifying diverse CHSs into different classes, regardless of their functional diversification, or on characterizing their functions in individual fungal species. A complete and systematic comparative analysis of CHS genes based on their orthologous relationships will be valuable for elucidating the evolution and functions of different CHS genes in fungi. Here, we identified and compared members of the CHS gene family across the fungal tree of life, including 18 divergent fungal lineages. Phylogenetic analysis revealed that the fungal CHS gene family is comprised of at least 10 ancestral orthologous clades, which have undergone multiple independent duplications and losses in different fungal lineages during evolution. Interestingly, one of these CHS clades (class III) was expanded in plant or animal pathogenic fungi belonging to different fungal lineages. Two clades (classes VI*b* and VI*c*) identified for the first time in this study occurred mainly in plant pathogenic fungi from Sordariomycetes and Dothideomycetes. Moreover, members of classes III and VI*b* were specifically up-regulated during plant infection, suggesting important roles in pathogenesis. In addition, CHS-associated networks conserved among plant pathogenic fungi are involved in various biological processes, including sexual reproduction and plant infection. We also identified specificity-determining sites, many of which are located at or adjacent to important structural and functional sites that are potentially responsible for functional divergence of different CHS classes. Overall, our results provide new insights into the evolution and function of members of CHS gene family in the fungal kingdom. Specificity-determining sites identified here may be attractive targets for further structural and experimental studies.

## Introduction

Chitin, a polymer of *N*-acetylglucosamine, is the second most abundant biomass in nature after cellulose (Merzendorfer, 2011). It is an important structural component of the cell wall in fungi, playing a crucial role in the maintenance of cell morphology (Bowman and Free, 2006). As a classic pathogen-associated molecular pattern (PAMP), fungal chitin can trigger innate immune responses in host plants and animals (Shibuya and Minami, 2001; Reese et al., 2007). A number of enzymes participate in the formation of chitin, but the chitin synthases (CHSs) are the crux of this process. CHSs are located in the plasma membrane and transfer *N*-acetylglucosamine to growing chitin chains (Merzendorfer, 2011). Owing to the lack of chitin in plants and mammals, CHSs are considered attractive targets for developing efficient antifungal agents used to control pathogenic fungi (Free, 2013).

CHS genes have been identified in various fungi. According to the phylogenetic positions and similarity in domain architecture, these genes were divided into three divisions (Roncero, 2002; Choquer et al., 2004; Mandel et al., 2006; Latgé, 2007). CHSs in division I contain type I (CS1, PF01644) and type II (CS2, PF03142) chitin synthase domains, as well as a chitin synthase N-terminal domain (CS1N, PF08407); CHSs in division II contain CS2 and other additional domains; CHSs in division III contain only the CS2 domain (Choquer et al., 2004; Mandel et al., 2006). Division I was further divided into three classes I, II, and III, and division II comprised three classes IV, V, and VII, whereas division III contained the single class VI (Mandel et al., 2006). Previous studies focused primarily on classifying diverse CHSs into different classes (Choquer et al., 2004; Mandel et al., 2006; Ruiz-Herrera and Ortiz-Castellanos, 2010). The majority of these studies employed a limited number of fungal species from a narrow range of fungal lineages (mostly later-branching fungi), which could lead to difficulty in establishing the relationships among different classes and even ambiguously classifying some CHSs into different or multiple classes. For example, class VI was clustered with division I in the study by Odenbach et al. (2009) but with division II in the study by Niño-Vega et al. (2004). In addition, some CHS genes from basidiomycetes of class II in the study of Ruiz-Herrera and Ortiz-Castellanos (2010) were classified into classes I and II in the study of Munro and Gow (2001). More recently, Pacheco-Arjona and Ramirez-Prado classified CHSs from 54 fungal genomes into the existing classification scheme (Pacheco-Arjona and Ramirez-Prado, 2014). However, the established classes of CHSs may contain distant ancestral paralogs with distinct functions in fungi. Because the orthologs retain the same function (Koonin, 2005), evolutionary classification of CHS genes based on the orthologous relationship is more reliable for transferring functional annotation.

Different CHSs have been proved to play various roles, suggesting that members of this family have undergone functional diversification to a certain extent. For example, the budding yeast *Saccharomyces cerevisiae* has three CHS genes with different functions. ScCHS1 replenishes chitin in the cell wall of daughter cells after cell division as a repair enzyme (Cabib et al., 1989). ScCHS2 participates in the processes of primary septum formation and cell division (Silverman et al., 1988). The majority of chitin is synthesized by ScCHS3, which is also responsible for forming the ring of chitin at the budding site (Shaw et al., 1991; Schmidt, 2004). Roles of CHSs are more complicated in filamentous fungi. In the plant pathogenic fungus *Magnaporthe oryzae*, for example, CHSs also participate in processes related to pathogenicity. The *Mochs1* deletion mutant has a defect in conidiogenesis, which limits the infection of host plants (Kong et al., 2012). MoCHS7 is responsible for appressorium penetration and invasive growth (Kong et al., 2012). Previous studies focused primarily on the functions of CHSs in individual fungal species. A complete and systematic comparative analysis of CHS gene family across the fungal kingdom will be valuable for elucidating their evolutionary and functional relationships.

In this study, we systematically identified members of the CHS gene family across 109 representative fungi from 18 major fungal lineages. The orthologous and paralogous relationships among various CHS genes were resolved. We found that one orthologous clade of CHS genes (class III) was expanded mainly in important animal or plant pathogenic fungi from different fungal lineages. We also report the identification of two novel clades of CHSs (classes VI*b* and VI*c*) that are present mainly in pathogenic fungi from Sordariomycetes and Dothideomycetes. Further expression analyses showed that members of classes III and VI*b* were specifically up-regulated during plant infection, suggesting important roles in pathogenesis. Moreover, we found that CHS-associated networks are conserved in plant pathogenic fungi and involved in various biological processes, including sexual reproduction and plant infection. In addition, we determined the specificity-determining sites that are potentially responsible for functional diversification of different CHS genes. Our results provide new insights into the evolution and function of the fungal CHS genes.

## Results and discussion

### The fungal CHS gene family is composed of at least 10 ancestral orthologous clades

**Figure 1:**
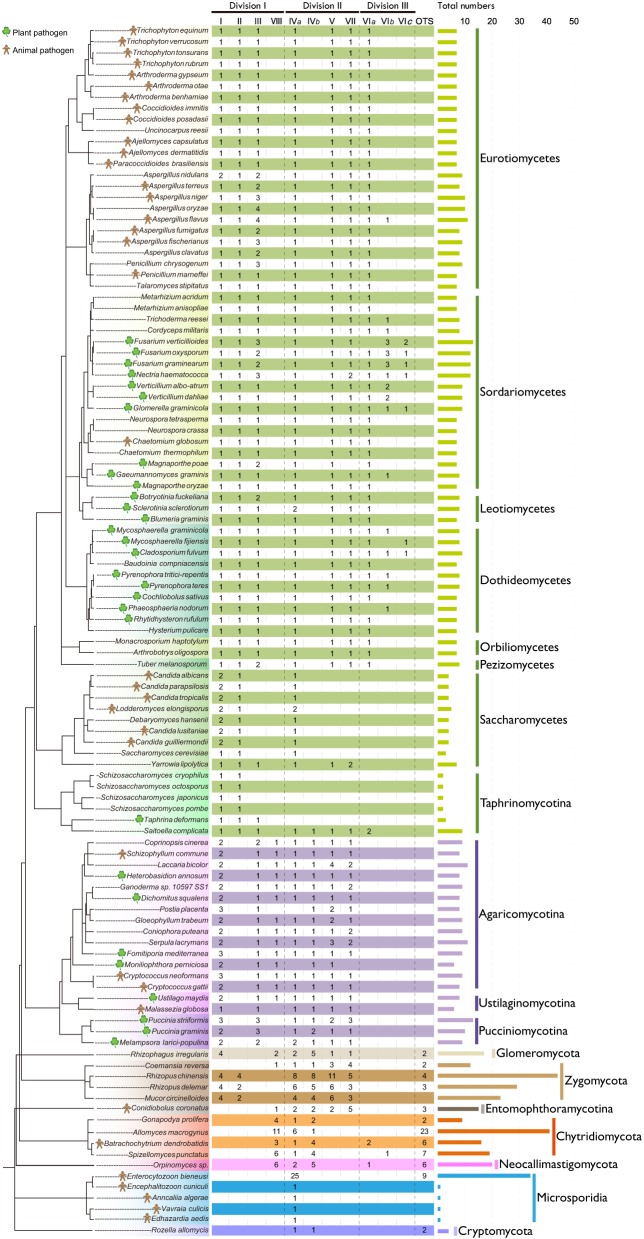
**Distribution of CHS genes across different fungal lineages**. The copy number of each orthologous CHS class is indicated. The species tree was constructed based on the phylogenetic tree of α-tubulins. The horizontal bar indicates the total number of CHSs in each fungus. For the definition of I, II, III, IV*a*, IV*b*, V, VII, VI*a*, VI*b*, VI*c*, VIII, and others (OTS), please see the text.

Based on the common conserved domains of CS1 and CS2, we identified 978 CHS genes in the predicted proteomes of 109 representative fungi from 18 fungal lineages in phyla Cryptomycota, Microsporidia, Neocallimastigomycota, Chytridiomycota, Entomophthoramycotina, Zygomycota, Glomeromycota, Ascomycota, and Basidiomycota (Figure 1; Table S1). Among these, 373 genes contained CS1N, CS1, and CS2 domains, suggesting that these genes were members of division I. Five additional genes lacked the CS2 domain and one of those also lacked the CS1N domain, which may be due to genome assembly gaps. The remaining 600 genes harbored only the CS2 domain, suggesting that these genes are members of division II or III.

**Figure 2:**
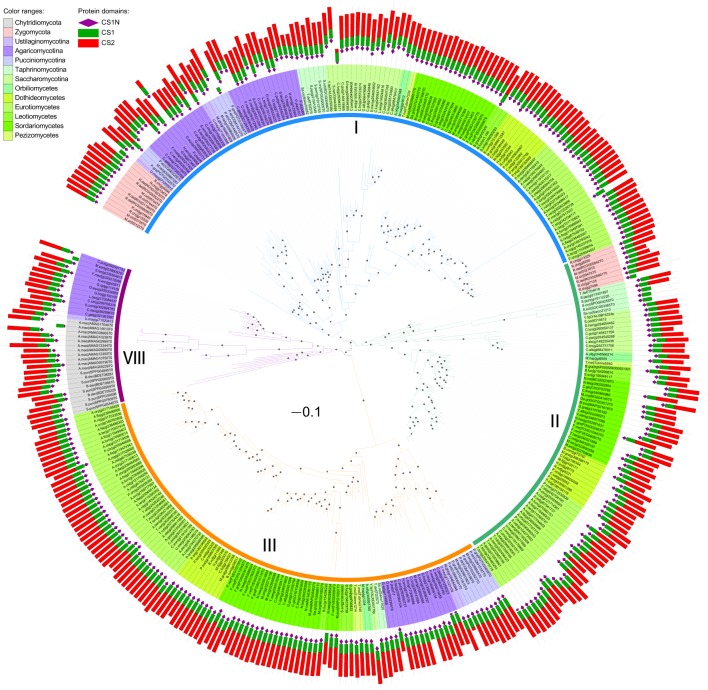
**Maximum likelihood phylogeny of division I CHSs**. The phylogenetic tree was constructed using PhyML3.1 (Guindon et al., 2010). The SH-like support of approximate likelihood ratios (aLRT-SH) are plotted as circles on the branches (only SH-like support >0.8 are indicated). Colors of branches indicate corresponding classes. The scale bar corresponds to 0.1 amino acid substitution per site. For abbreviations of fungi, please see Table S1.

We then performed maximum likelihood phylogenetic analyses to determine the orthologous and paralogous relationships of these identified CHS genes based on their common conserved domains. The phylogenetic tree of division I displayed four distinct ancestral orthologous clades (Figures 2,**4A**). Three of these orthologous clades correspond to classes I, II and III defined previously (Munro and Gow, 2001; Choquer et al., 2004). Therefore, we define them as classes I, II and III, respectively. Notably, previous studies showed ambiguous phylogenetic positions of classes I and II CHS genes from basidiomycetes (Munro and Gow, 2001; Pacheco-Arjona and Ramirez-Prado, 2014), but our study clearly suggested that these CHS genes should be within class I. A fourth orthologous clade contained CHS genes from basidiomycetes and early-branching fungal lineages, including Neocallimastigomycota, Chytridiomycota, Zygomycota, and Glomeromycota. This clade was identified for the first time in this study and defined as class VIII.

**Figure 3:**
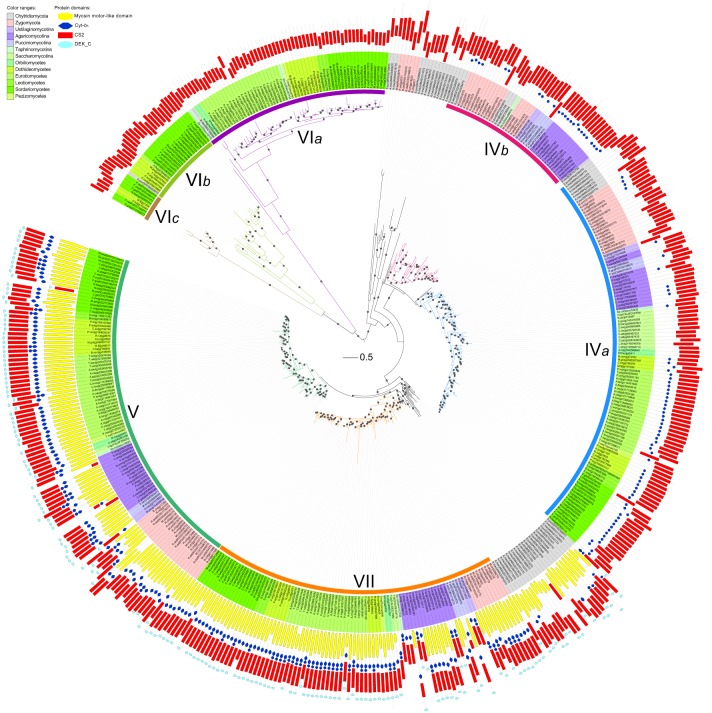
**Maximum likelihood phylogeny of divisions II and III CHSs**. The phylogenetic tree was constructed using PhyML3.1. The SH-like support of approximate likelihood ratios (aLRT-SH) are plotted as circles on the branches (only SH-like support >0.8 are indicated). Colors of branches indicate corresponding classes. The scale bar corresponds to 0.5 amino acid substitution per site. For abbreviations of fungi, please see Table S1.

**Figure 4:**
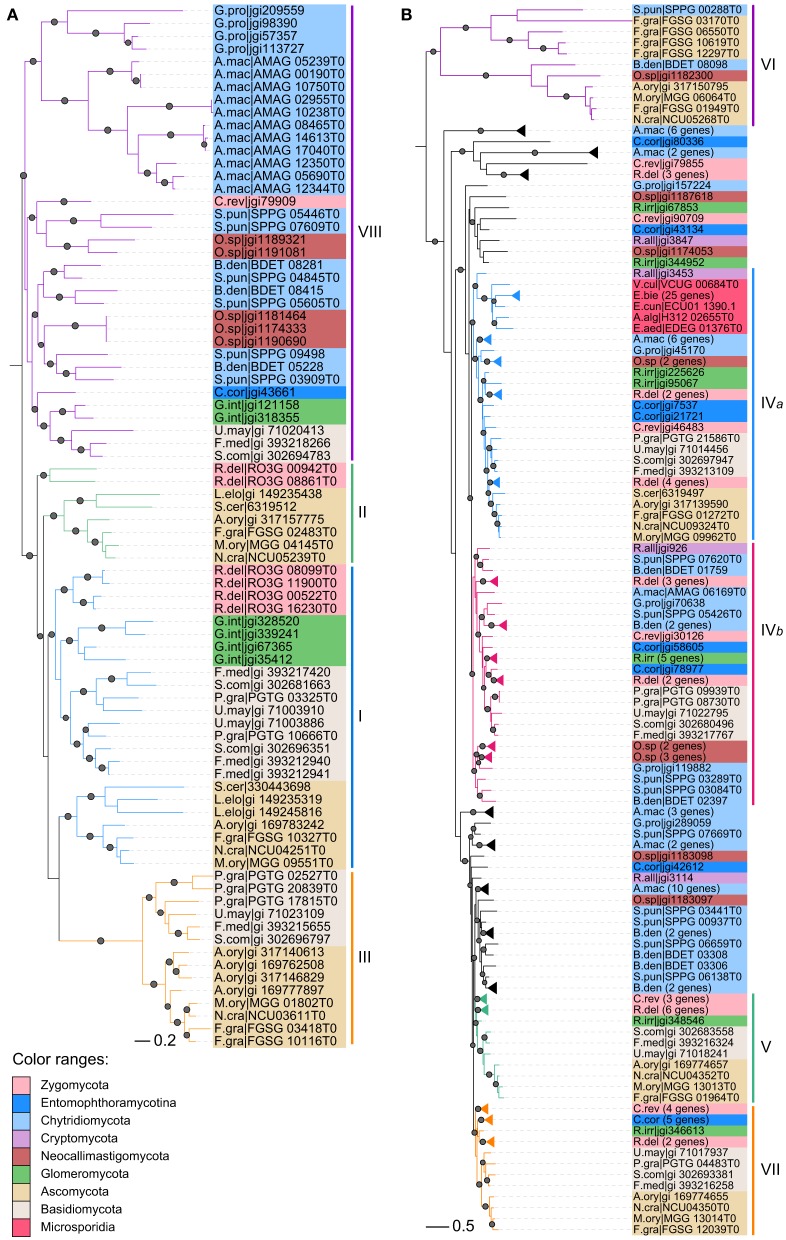
**Maximum likelihood phylogenies of CHS genes from early-branching fungi**. **(A)** Division I. **(B)** Divisions II and III. Phylogenetic tree was constructed using PhyML3.1. The SH-like support of approximate likelihood ratios (aLRT-SH) are plotted as circles on the branches (only SH-like support >0.8 are indicated). Colors of branches indicate corresponding classes. For abbreviations of fungi, please see Table S1.

Phylogenetic analysis showed that members of divisions II and III are distantly related to each other (Figures 3,4B). Division II is comprised of four distinct orthologous clades. Two clades correspond to classes V and VII defined previously (Mandel et al., 2006) and are designated as classes V and VII in this study. Notably, class IV defined previously (Munro and Gow, 2001; Choquer et al., 2004; Pacheco-Arjona and Ramirez-Prado, 2014) was actually comprised of two orthologous clades: classes IV*a* and IV*b*. Orthologous relationships of some CHS genes from early-branching fungi were not determined. These genes were temporarily labeled as “others” (OTS) (Figure 1). Division III is comprised of three distinct orthologous clades. One of them (class VI*a*) corresponds to class VI defined previously (Munro and Gow, 2001). The other two orthologous clades (classes VI*b* and VI*c*) are reported for the first time in this study.

Comparison of sequence identity of CHSs showed that members in the same class are more closely related to each other, but distantly related to members in other classes (Figure S1). Each class formed a distinct group. These results are consistent with those of our phylogenetic analysis. CHSs of division III are more similar to those of division II than division I.

### Domain architectures of CHSs are consistent in divisions I and III but diverse in division II

Besides the three CS1N, CS1, and CS2 domains, no additional domains were identified in CHSs of division I (Figure 2). Likewise, no other domain was found in CHSs of division III besides the CS2 domain, but most CHSs of division II also contain the Cytochrome b5-like (Cyt-*b*_5_, PF00173) domain (Figure 3). This domain is absent from some members of classes IV*a* and IV*b* in division II. The Cyt-*b*_5_ domain can bind heme and steroids in electron transfer (Schenkman and Jansson, 2003), but the ligand for this domain in fungal CHSs is unknown. An N-terminal myosin motor-like domain (MMD, PF00063) and a DEK C-terminal (DEK_C, PF08766) domain also exist in classes V and VII CHSs. The MMD assists the localization of CHSs to the hyphae via interaction with the actin cytoskeleton, and facilitates polarized exocytosis (Tsuizaki et al., 2009; Schuster et al., 2012). The DEK_C domain is related to the C-terminal of animal DEK protein, which is responsible for self-multimerization and DNA binding (Kappes et al., 2004). Nevertheless, the role of the DEK_C domain in fungi remains to be determined.

### Variable numbers of CHSs in different fungal lineages

Yeasts generally contain few CHSs (Figure 1). For example, the fission yeasts possess only two CHSs, the fewest copies among all fungal lineages, which may be related to the absence of chitin in their vegetative cell walls (Free, 2013). The budding yeast *S. cerevisiae* harbors three CHSs. Filamentous fungi usually contain a larger number of CHSs. For example, the cereal pathogen *Fusarium verticillioides* has 13 CHSs, the highest number of copies among the later-branching fungi, ascomycetes and basidiomycetes. On the other hand, the early-branching fungi, chytridiomycetes, entomophthoromycetes, zygomycetes, and glomeromycetes, generally possess more CHSs than the later-branching fungi (Figure 1), likely because the cell walls of early-branching fungi contain a higher percentage of chitin than those of later-branching fungi (Ma et al., 2009). Unexpectedly, the zygomycete *Rhizopus chinensis* has 44 CHS genes, the largest number of CHS genes discovered to date. Microsporidia generally contain one CHSs, but *Enterocytozoon bieneusi* has many more CHS genes than other species in this lineage, consistent with a previous report (Pacheco-Arjona and Ramirez-Prado, 2014).

### Frequent duplications and losses of CHS genes in different fungal lineages

The classes IV*a*, IV*b*, VI*a*, VI*b*, and VIII occur in both early-branching fungi and later-branching fungi (Figure 1), suggesting that they were generated in fungal ancestors. However, during subsequent evolution, the class VIII was lost in all ascomycetes and most zygomycetes. In addition, the class III was lost in all early-branching fungal lineages, in agreement with previous reports (Ruiz-Herrera and Ortiz-Castellanos, 2010; Pacheco-Arjona and Ramirez-Prado, 2014). The class IV*b* was lost in all ascomycetes but not in the archiascomycetous yeast *Saitoella complicata*. The class IV*a* was lost in individual fungal lineages, such as fission yeasts. The classes VI*a* and VI*b* were lost in basidiomycetes and most of early-branching fungi. Unexpectedly, in contrast to other yeasts that retained only the classes I, II, and IV*a, Yarrowia lipolytica* and *S. complicata* contained most of the orthologous classes.

In addition to gene losses, duplications of the CHS genes occurred frequently in different fungal lineages during evolution. For example, members of class I were duplicated in Basidiomycota, Zygomycota, and *Candida* species. CHS genes in each class were duplicated multiple times in early-branching fungi, and those from one species generally clustered together (Figures 2–4), suggesting that they were derived from species-specific expansions. In fact, the genome of *Rhizopus delemar* underwent a whole-genome duplication and recent gene duplications (Ma et al., 2009).

Notably, duplications in members of class III were most common in plant or animal pathogens within *Aspergillus* spp., *Fusarium* spp., and Pucciniomycotina. Since the class III CHS has been experimentally demonstrated to play important roles during host infection in the rice blast fungus *M. oryzae* (Kong et al., 2012), the expansion of this class in pathogenic fungi may be related to infection and pathogenicity of these fungi. Furthermore, the classes VI*b* and VI*c* CHSs occurred mainly in plant pathogens from Sordariomycetes and Dothideomycetes.

### Specificity-determining sites responsible for functional divergence in the CHS gene family

Previous studies have revealed that CHSs of several classes perform different functions in fungi (Silverman et al., 1988; Cabib et al., 1989; Shaw et al., 1991; Kong et al., 2012). It is known that functional divergence commonly results from the shifts at specificity-determining sites (Gu, 2001). Therefore, we examined CS domains among the orthologous classes for functional specificity-determining sites.

**Figure 5:**
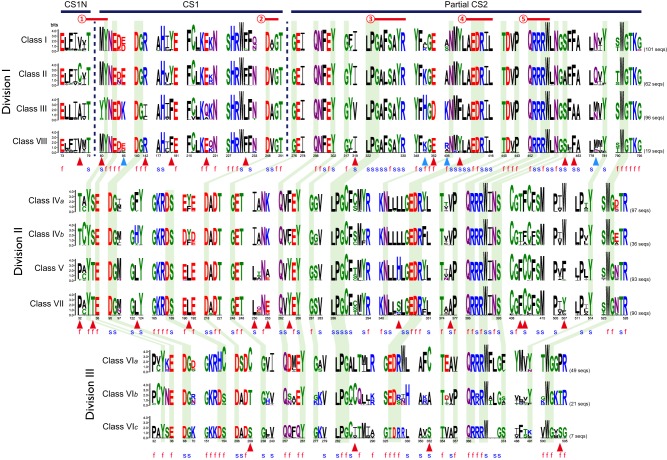
**Conservation and diversification of CS domains in the fungal CHS gene family**. Sequence logos indicate conserved sequence patterns of CHS domain in each class. Black horizontal bars indicate regions of domains that are defined by the Pfam database. Red horizontal lines indicate conserved functional motifs according to the CDD database of NCBI and the previous study (Zakrzewski et al., 2014): ➀, ligand binding; ➁, metal ion binding site; ➂, donor saccharide binding; ➃, acceptor saccharide binding; ➄, product binding. Green shaded regions represent sites that are conserved across all classes. Red and blue triangles indicate type I and type II specificity-determining sites identified by SPEER-SERVER (Chakraborty et al., 2012), respectively. The letters f and s represent functional sites and structural sites identified by ConSurf (Ashkenazy et al., 2010), respectively.

In division I, we identified 15 specificity-determining sites (Figure 5). Four of them are likely related to type I functional divergence. Residues at these sites are conserved in one orthologous clade but highly variable in the other clades, indicating that functional constraints have changed between them (Gu, 2001). The remaining 11 sites are related to type II functional divergence. Residues at these sites are conserved in different clades but their properties are dissimilar, which could lead to functional specifications within a gene family (Gu, 2001). Ten specificity-determining sites are potentially responsible for the functional divergence between class III and the other three classes. In addition, the residue at site 180 is Phe (F) in classes III and VIII, while the corresponding residue in classes I and II is Tyr (Y). Functional divergence of class II from the other three classes may be due to a shift at site 460 from Ser (S) to Ala (A). In division II, we identified 12 type II specificity-determining sites. Residues at all of these sites in classes IV*a* and IV*b* are distinct from those in classes V and VII. At site 123, functional divergence of class IV*a* from class IV*b* may be due to a shift from His (H) to Phe (F). At site 344, functional divergence of class V from class VII may result from a Ser (S) to His (H) shift. In division III, we identified four type II specificity-determining sites. Residues at these sites differ among the three classes. For example, the residues at site 209 are Cys (C), Thr (T), and Ser (S) in classes VI*a*, VI*b*, and VI*c*, respectively.

Most of the specificity-determining sites are adjacent to or located at the functionally or structurally important sites of CHSs identified by ConSurf (Figure 5; Figures S2–S4; Ashkenazy et al., 2010). For example, in division I, type II specificity-determining site 80 is directly involved in ligand binding (Figure 5). This shift probably affects the chemical conformation of CHSs. Additionally, in division II, the structural site 346, which may have undergone type II functional divergence from division I, is adjacent to the acceptor saccharide binding sites. This shift may affect the interaction between CHSs and acceptors. Previous studies have experimentally demonstrated two functionally important motifs EDRXL and QRRRW of CHSs that are responsible for acceptor saccharide binding and product binding in yeast (Nagahashi et al., 1995; Choquer et al., 2004; Zakrzewski et al., 2014). These two motifs are well conserved in all classes of CHSs, confirming their critical roles. Interestingly, four functional specificity-determining sites are adjacent to these two motifs. The site 409 upstream three residues of the EDRXL motif underwent alteration in class III CHSs from Tyr (Y) to Phe (F). Likewise, the site 462 downstream six residues of the QRRRW motif underwent alteration in class III CHSs from Phe (F) to Ala (A). These sites may be responsible for functional divergence of classes III and the other three classes of division I.

### Members of classes III and VI*b* CHS genes are specifically up-regulated during infection

**Figure 6:**
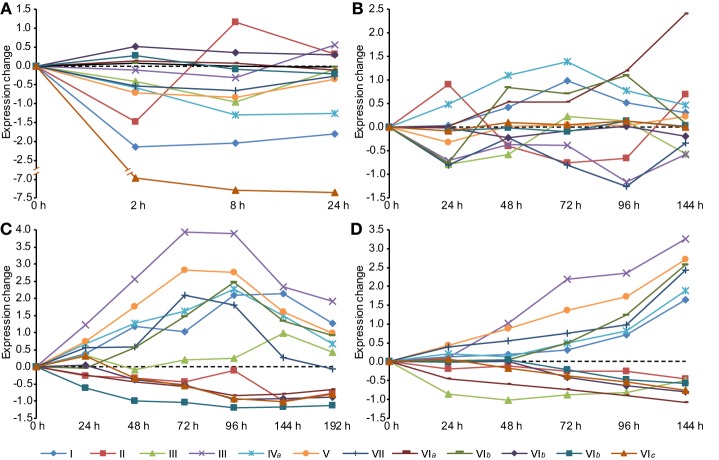
**Expression profiles of CHS genes during different stages in ***Fusarium graminearum*****. **(A)** Conidia germination. **(B)** Sexual development *in vitro*. **(C)** Infection of wheat. **(D)** Infection of barley spikes. In each condition, the mean values of RMA normalized expression data of three independent biological duplicates were analyzed. All time-series were transformed to (0, v_1-v_0, v_1-v_0,…, v_n-v_0) so that the time series begins at 0.

Because classes III, VI*b*, and VI*c* were mainly retained or expanded in important pathogenic fungi, we examined the expression of these CHSs during infection. In the wheat scab fungus *Fusarium graminearum*, one of two paralogs of class III CHS genes (FGSG_10116) was specifically up-regulated during spike infection of barley and wheat (Figure 6). This class III gene had the highest expression change during host infection relative to other CHS genes, in agreement with the results of a recent study (Cheng et al., 2015). Deletion of this gene was lethal to *F. graminearum* (Cheng et al., 2015). Its ortholog in *M. oryzae* (MGG_01802) was responsible for virulence (Kong et al., 2012). In the stem rust fungus *Puccinia graminis* f. sp*. tritici*, one of the three copies (PGTG_02527) of class III CHSs was also highly induced during infection of wheat and barley (Figure S5A). These results indicate that the orthologous class III in pathogenic fungi may perform important functions during host infection.

One CHS gene of class VI*b* (FGSG_06550) from *F. graminearum* was highly induced during the later phases of infection (Figure 6). Its ortholog (GI: 310801203) in the maize anthracnose fungus *Glomerella graminicola* was also up-regulated during infection and had the highest expression level in the necrotrophic phase among all CHSs (Figure S5B), suggesting that this class VI*b* CHS is important for pathogenesis.

In addition, genes in classes V (FGSG_01964) and VII (FGSG_12039) were highly induced during host infection. Previous studies have demonstrated that the classes V and/or VII CHSs are required for pathogenicity in many plant pathogens, such as *F. graminearum, F. verticillioides, Fusarium oxysporum, M. oryzae, G*. *graminicola*, and *Ustilago maydis* (Madrid et al., 2003; Garcerá-Teruel et al., 2004; Werner et al., 2007; Martín-Urdíroz et al., 2008; Kim et al., 2009; Treitschke et al., 2010; Larson et al., 2011; Kong et al., 2012).

### The CHS-associated network is conserved in pathogenic fungi

**Figure 7:**
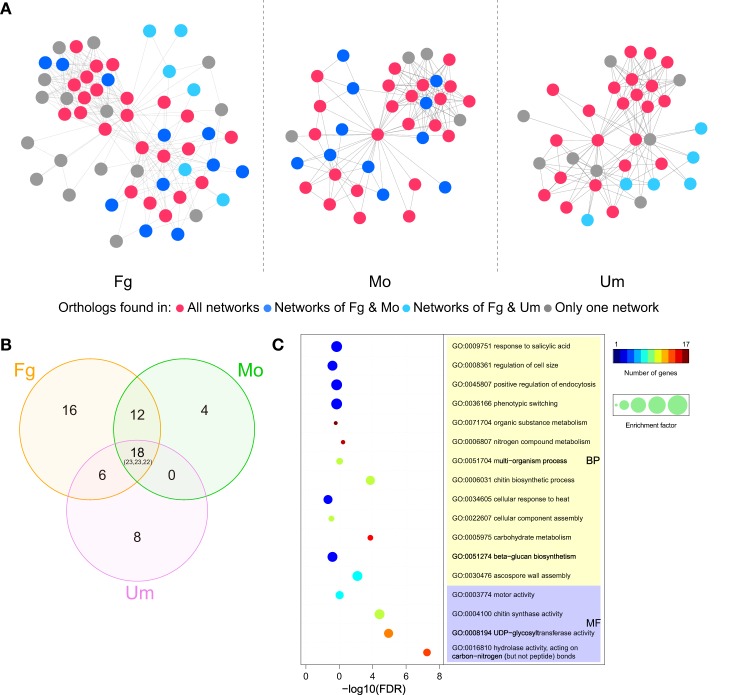
**CHS-associated networks in ***Fusarium graminearum, Magnaporthe oryzae***, and ***Ustilago maydis*****. **(A)** CHS-associated networks in three plant pathogens. Nodes and edges represent genes and functional links, respectively. Node colors indicate the range of ortholog groups that appear in three networks. **(B)** Venn diagram for the distribution of ortholog groups in the three networks. Numbers correspond to ortholog groups, while the numbers in brackets represent the total number of genes (Ordering: Fg-Mo-Um). **(C)** Selected GO terms enriched in ortholog genes that are conserved in the three networks. The circle colors indicate the number of genes with a relevant GO term in the test set. The circle size indicates the ratio of the percentage of genes with relevant GO terms in the test set to that in the reference set (enrichment factor). Fg, *F. graminearum*; Mo, *M. oryzae*; Um, *U. maydis*. Please see Tables S2, S3 for more detailed information.

To better understand the functional conservation of CHSs in pathogenic fungi, we compared functional protein-associated networks involved in chitin biosynthesis, constructed based on co-expression evidence and experimental evidence from the STRING database (Szklarczyk et al., 2015) in three important pathogenic fungi. A total of 57, 39, and 36 genes were determined to be functionally associated with the CHSs in *F. graminearum, M. oryzae*, and *U. maydis*, respectively (Figure 7A; Table S2). In the three CHS-associated networks, about half of the genes were conserved (Figures 7A,B). Gene ontology (GO) enrichment analysis showed that, beyond chitin biosynthesis, genes involved in ascospore formation, phenotypic switching, positive regulation of endocytosis, and response to salicylic acid were significantly enriched in these conserved orthologous genes (Figure 7C; Table S3). These results indicated that the CHS-associated network is likely related to fungal pathogenicity. In *F. graminearum*, ascospores are the primary inoculum for infecting wheat and barley (Trail et al., 2002). In addition, salicylic acid (SA) is considered to be a key plant defense hormone. A previous study demonstrated that *U. maydis* could sense and restrict the SA-levels of host plants to assure colonization success (Rabe et al., 2013).

**Figure 8:**
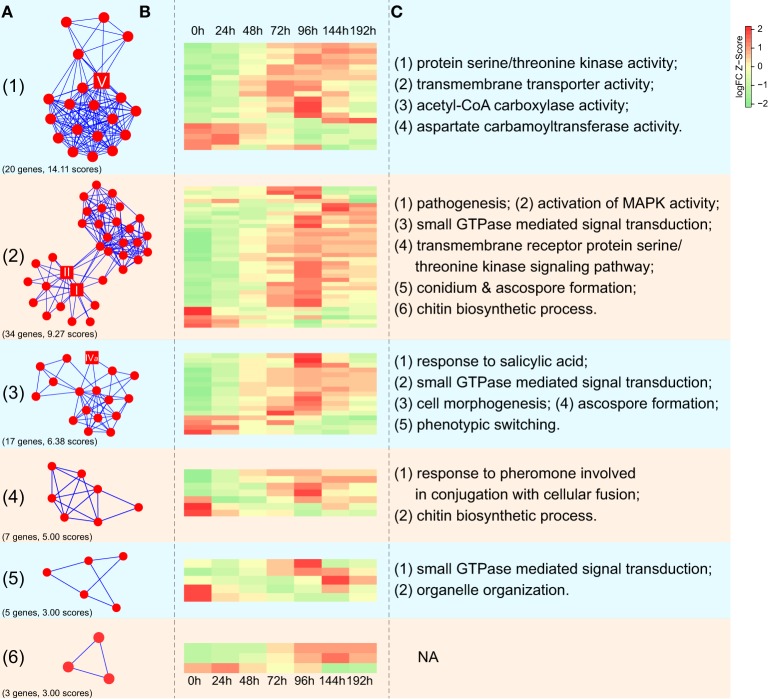
**Expression profiles of interaction clusters in CHS-associated networks in ***F. graminearum*****. **(A)** Interaction clusters identified in the network of CHSs in *F. graminearum*. The square nodes represent CHSs of *F. graminearum*. Numbers in the brackets correspond to gene numbers and scores calculated by MCODE (Bader and Hogue, 2003), respectively. Please see Table S4 for more detailed information. **(B)** Dynamic expression profiling of interaction clusters during infection of wheat. Each row in the heatmap indicates a single gene and each column represents a time point after wheat was inoculated with *F. graminearum*. The scale bar represents the Z-scores of log_2_-fold change. **(C)** Selected GO terms enriched in five interaction clusters. Please see Table S5 for more detailed information.

We identified the putative protein complexes or parts of pathways hidden in the CHS network of *F. graminearum* constructed from the evidence of genomic context, experiments and text-mining. Among the 171 genes in the network, six interaction clusters were detected and found to be comprised of 20, 34, 17, 7, 5, and 3 genes, respectively (Figure 8A; Table S4). Based on the expression data downloaded from PLEXdb (Dash et al., 2012), most of the genes in these clusters were up-regulated during the later phases (72, 96, or 144 h) of wheat infection. Although some genes exhibited the opposite expression pattern, the entire clusters seemed to be co-regulated. For example, contrary to most genes in cluster 2 that were induced along the timeline, five genes in the same cluster were repressed (Figure 8B). These results suggested that genes in these clusters might play important roles in the necrotrophic phase of *F. graminearum*. Further functional enrichment analysis indicated that genes in cluster 2 are involved in various processes, such as pathogenesis, the MAPK cascade, small GTPase-mediated signal transduction, transmembrane receptor protein serine/threonine kinase signaling pathway, and ascospore formation (Figure 8C; Table S5). Many of these processes are related to fungal pathogenicity (Bölker, 1998; Xu et al., 1998; Beyer and Verreet, 2005). In addition, cluster I includes acetyl-CoA carboxylase and aspartate carbamoyltransferase, which are essential for the survival of fungi due to their crucial roles in the biosynthesis of fatty acids and pyrimidines (Simmer et al., 1990; Tong, 2005). Furthermore, calcineurin subunit B (FGSG_07404) in cluster 4 is involved in the adaptation to pheromone during conjugation with cellular fusion, which is essential for normal vegetative growth in *Neurospora crassa* (Kothe and Free, 1998).

## Conclusions

We systematically identified and compared CHS genes across 109 representative fungi from Ascomycota, Basidiomycota, Cryptomycota, Microsporidia, Chytridiomycota, Zygomycota, Neocallimastigomycota, Entomophthoramycotina, and Glomeromycota. Comparative analysis revealed diversity in the number of CHS genes among different fungal lineages. Phylogenetic analysis revealed that the fungal CHS gene family is comprised of at least 10 ancestral orthologous clades, which have undergone frequent duplications and losses in different fungal lineages during evolution. Our results showed that CHS genes in classes III and VI*b* have expanded or have been retained in pathogenic fungi and that they likely perform important functions during host infection. Comparative analysis revealed that CHS-associated networks are conserved among important pathogenic fungi, and participate in various important processes, including sexual reproduction and plant infection. In addition, many specificity-determining sites are located at or adjacent to important sites such as ligand binding and acceptor saccharide sites of CHSs and probably lead to functional diversification in the CHS gene family. These specificity-determining sites may be attractive targets for further structural and experimental studies. Results from this study elucidated orthologous and paralogous relationships among various CHS genes and provided new insights into the evolution and function of the fungal CHS genes.

## Materials and methods

### Collection of fungal genomes

The predicted proteomes of 109 public fungal genomes (Table S1) were collected from GenBank of National Center for Biotechnology Information (NCBI), Joint Genome Institute (JGI) site of DOE (http://genome.jgi.doe.gov/programs/fungi/index.jsf), Fungal Genome Initiative (FGI) site of Broad Institute (http://www.broadinstitute.org/science/projects/projects/fungal-genome-initiative), UniProt database (http://www.uniprot.org/proteomes/), and Blugen (http://www.blugen.org/).

### Identification of putative CHSs in fungi

The Hmmscan program in HMMER 3.0 package (Eddy, 2011) was used to search each of the fungal proteomes with the domain-specific HMM profiles of chitin synthases downloaded from the Pfam database (Finn et al., 2014) as queries, including Chitin_synth_1 (PF01644) and Chitin_synth_2 (PF03142) domains. The primary result was filtered with the score of 20 as the cutoff. Only the sequences marked as CHSs (best match) were submitted to the Pfam database and the SMART database (Letunic et al., 2012) to confirm the chitin synthase domains.

### Sequence alignment and phylogenetic analysis

Multiple sequence alignments were generated with the M-Coffee program (Di Tommaso et al., 2011) using the combination of T-Coffee, MAFFT, MUSCLE, and ProbCons methods and further edited manually. CHSs in division I were aligned based on the continuous CS1N, CS1, and CS2 domains, whereas CHSs in divisions II and III were aligned based only on the CS2 domain. Phylogenies were subsequently constructed by the Maximum likelihood (ML) method using PhyML3.1 (Guindon et al., 2010) with eight categories of γ-distributed substitution rates and SPRs algorithms, based on the best-fit model LG + G estimated by ProtTest2.4 (Abascal et al., 2005). SH-like approximate likelihood ratios (aLRT-SH) supports were used to evaluate the reliability of internal branches. The trees were further edited using the ITOL tool (Letunic and Bork, 2007). The identity scores of alignment were extracted with BioEdit software and then the heat map was constructed with the gplot package in R.

### Prediction of specificity-determining sites in the fungal CHS gene family

Complete sequences of CHSs in each division were aligned using COBALT (Papadopoulos and Agarwala, 2007). Specificity-determining sites were identified using SPEER-SERVER (http://www.hpppi.iicb.res.in/ss/index.html) with highest scores. The options of 20% gap allowed per column and a weight of 1.0 for relative entropy and physico-chemical (PC) properties were used (Chakraborty et al., 2012). A sequence logo of each class of CHSs was created with WebLogo 3 (Crooks et al., 2004). Structural sites and functional sites were identified using the ConSurf Server (Ashkenazy et al., 2010). The default Bayesian calculation method and JTT evolutionary substitution model were used.

### Analysis of gene expression profiles of CHSs in plant pathogens

The mean values of Robust Multi-array Average (RMA) normalized expression data of *F. graminearum* were obtained from the Plant Expression Database (PLEXdb) (http://www.plexdb.org/index.php) with four individual experiments, including conidia germination (Experiment FG7), sexual development (Experiment FG5) and spike infection of barley (Experiment FG1) and wheat (Experiment FG15). For each experiment, all time-series were transformed to (0, v_1-v_0,…, v_n-v_0) so that the time series begins at 0. The normalized gene expression data of *G. graminicola* (O'Connell et al., 2012) and *P. graminis* f. sp. *tritici* (Duplessis et al., 2011) were downloaded from NCBI GEO (accession no. GSE34632 and GSE25020). The data of three biological replicates were averaged and further transformed to log_2_ ratios.

### Network analysis

We used CHSs as baits to extract all the functional protein-associated networks using different evidence in the STRING (version 10) database (Szklarczyk et al., 2015) with a confidence score of 0.4. Then CHS-associated networks were visualized in Cytoscape (version 3.2.0) software (Shannon et al., 2003). Interaction clusters were detected using MCODE (version 1.4.1) plugin (Bader and Hogue, 2003) with the default parameters. Functional enrichment analysis was performed using Ontologizer 2.1 software (Bauer et al., 2008) with the whole *F. graminearum* annotation as the reference set. The term-for-term approach and Benjamini and Hochberg FDR correction (adjusted *p* < 0.05) options were used. Heatmaps were constructed with the R package gplot. Hierarchical clustering was carried out with Pearson correlation distance measure and the pairwise average-linkage method.

## Author contributions

JRX conceived the study; HL designed and coordinated the study; ML and CJ performed bioinformatic analyses; QW and ZZ contributed to the data collection; ML, CJ, QW, QJ, JX, and HL interpreted the results; ML and HL wrote the paper. All authors read, corrected and approved the final manuscript.

### Conflict of interest statement

The authors declare that the research was conducted in the absence of any commercial or financial relationships that could be construed as a potential conflict of interest.
