# Supplementary material for: Evolution and Functional Insights of Different Ancestral Orthologous Clades of Chitin Synthase Genes in the Fungal Tree of Life
Source: Front Plant Sci. 2016 Feb 1;7:37. doi: 10.3389/fpls.2016.00037 (PMC4734345; doi:10.3389/fpls.2016.00037)
Supplement: Supplementary file 6 [file Image1.PDF]

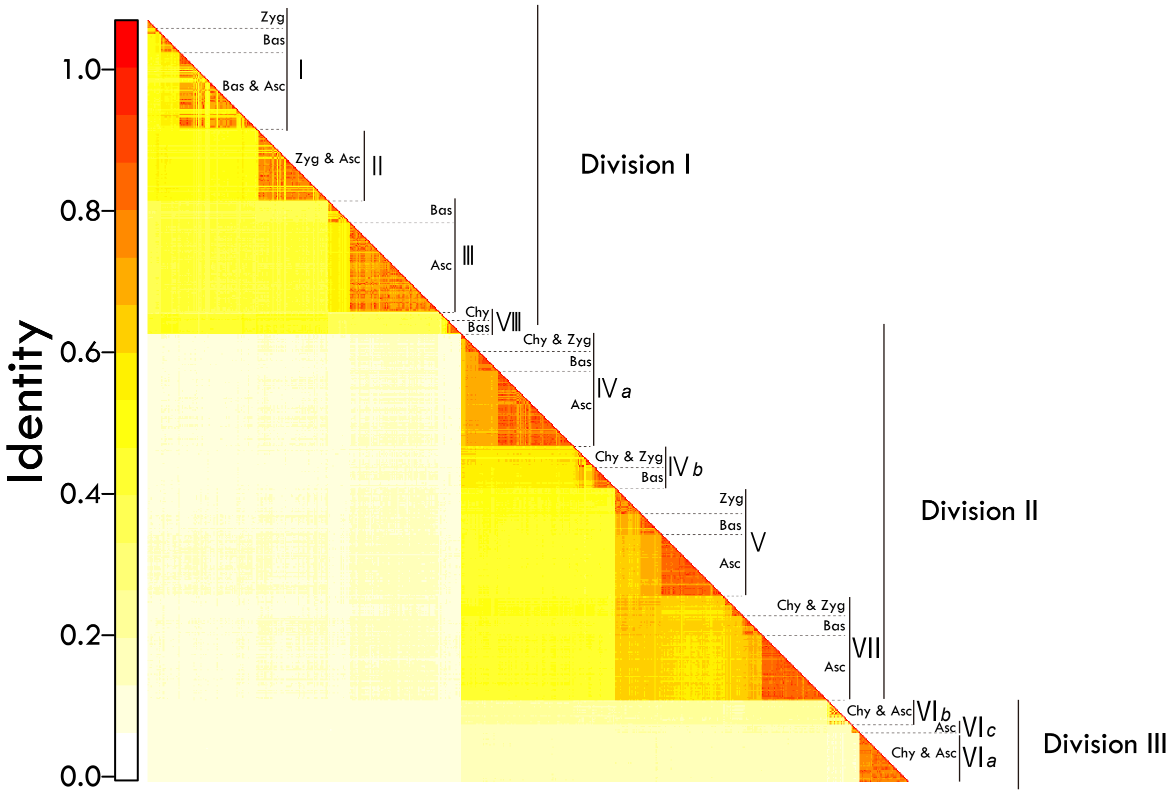

Figure S1. Heatmap of identity matrix. The scale bar represents identity scores. Abbreviated taxa are: Zyg, *Zygomycota*; Chy, *Chytridiomycota*; Asc, *Ascomycota*; Bas, *Basidiomycota*. See the text for the definition of different CHS classes (I – VIII).
