# Supplementary figures and images for: Evolution and Functional Insights of Different Ancestral Orthologous Clades of Chitin Synthase Genes in the Fungal Tree of Life
Source: Front Plant Sci. 2016 Feb 1;7:37. doi: 10.3389/fpls.2016.00037 (PMC4734345; doi:10.3389/fpls.2016.00037)

Vib

Vlc

Vla

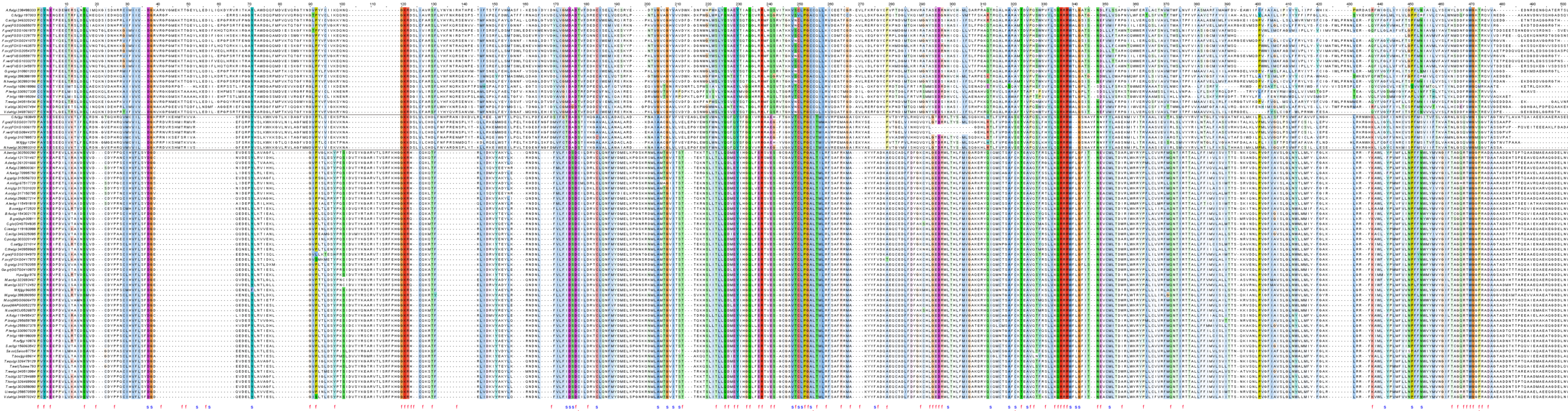

Supplement: Supplementary file 9 [file Image4.pdf]
