# Supplementary material for: Evolution and Functional Insights of Different Ancestral Orthologous Clades of Chitin Synthase Genes in the Fungal Tree of Life
Source: Front Plant Sci. 2016 Feb 1;7:37. doi: 10.3389/fpls.2016.00037 (PMC4734345; doi:10.3389/fpls.2016.00037)
Supplement: Supplementary file 10 [file Image5.PDF]

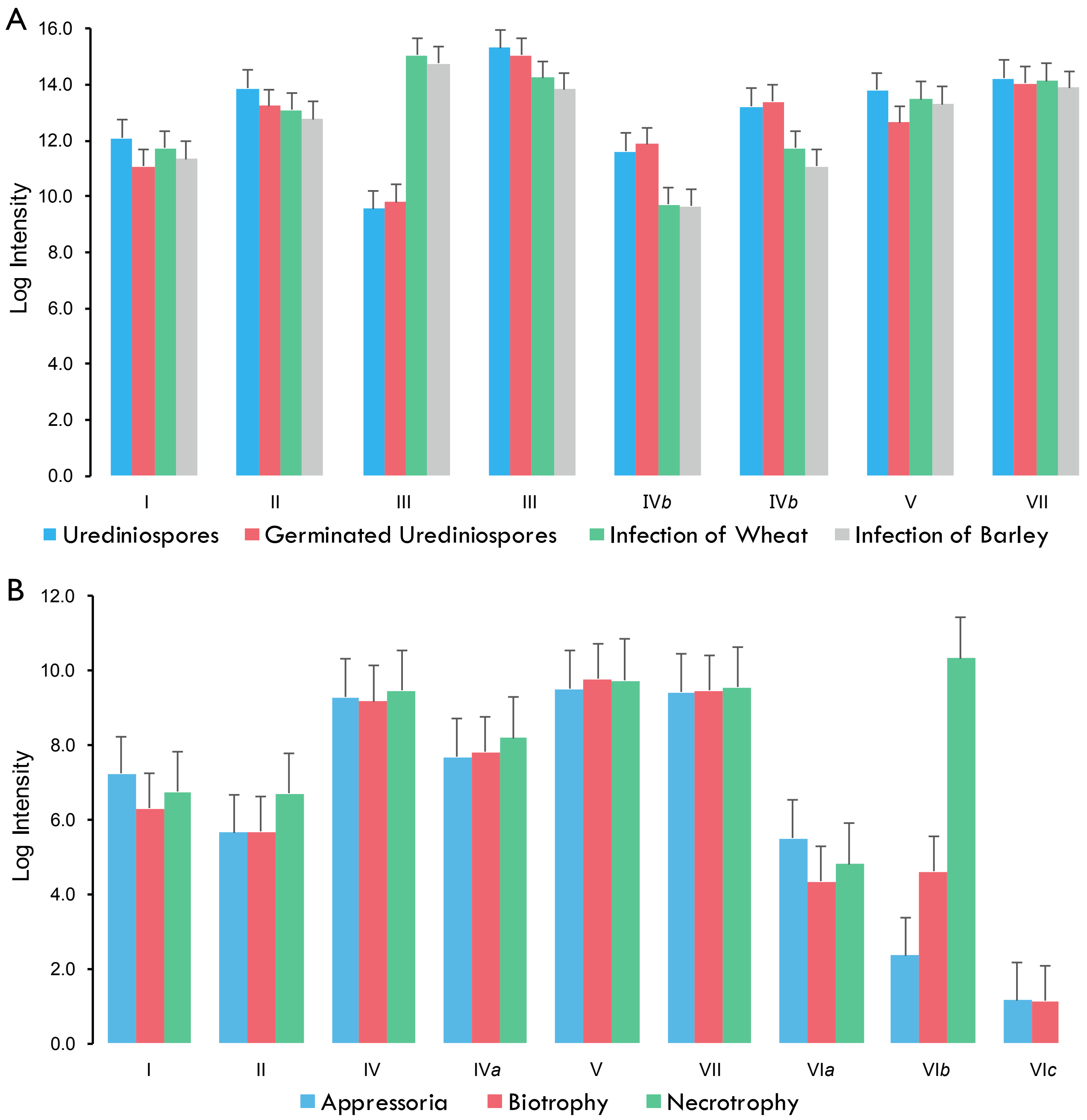

Figure S5. **Expression levels of CHSs at different stages in *Puccinia graminis* f. sp. *tritici* and *Glomerella graminicola*.** The bars represent mean log2 ratio values ± standard error of three independent biological experiments. **(A)** Expression profiles of CHSs at four developmental stages of *P. graminis* f. sp. *tritici*. **(B)** Expression profiles of CHSs at three developmental stages of *G. graminicola* during infection of Zea mays leaf sheaths.
