# Supplementary material for: Evolution and Functional Insights of Different Ancestral Orthologous Clades of Chitin Synthase Genes in the Fungal Tree of Life
Source: Front Plant Sci. 2016 Feb 1;7:37. doi: 10.3389/fpls.2016.00037 (PMC4734345; doi:10.3389/fpls.2016.00037)
Supplement: Supplementary file 7 [file Image2.PDF]

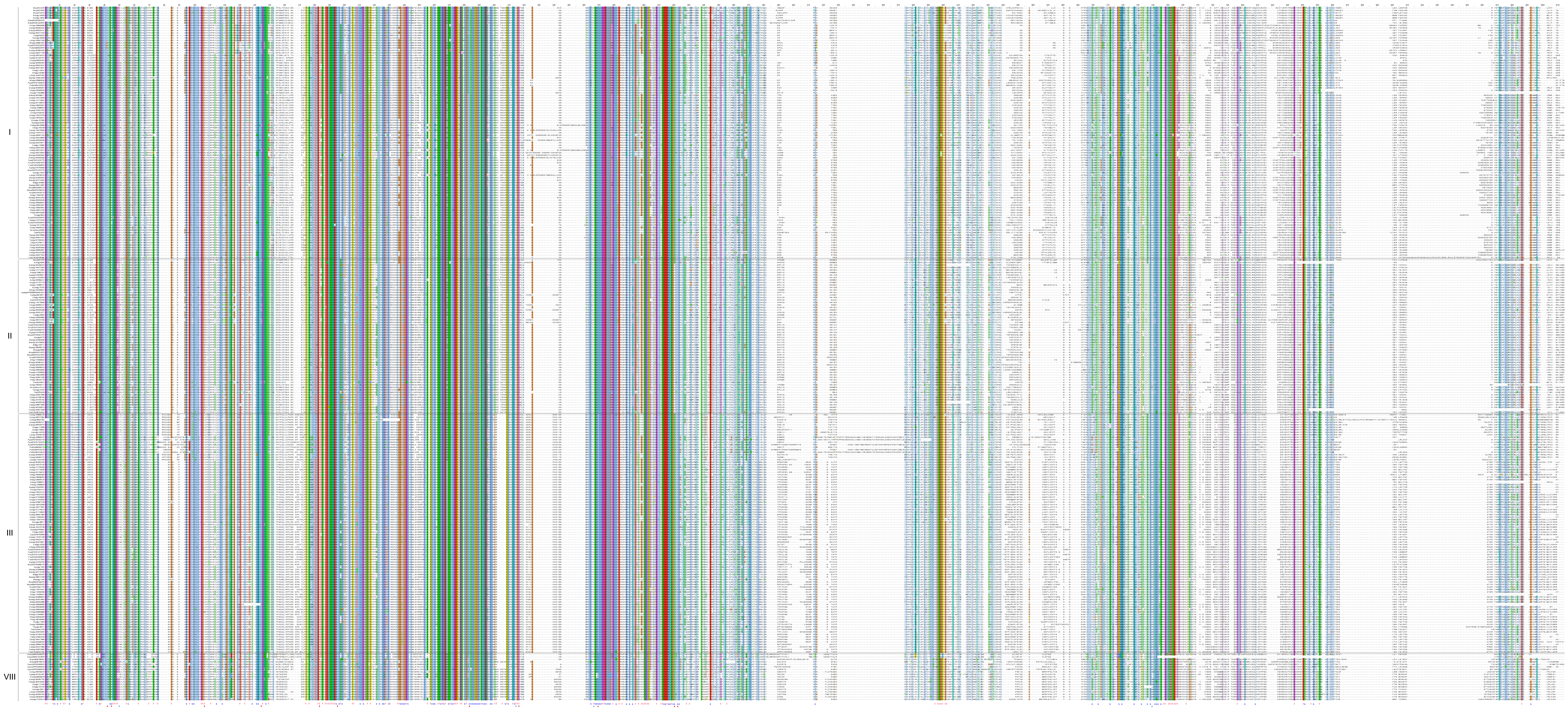

Figure S2. Multiple sequence alignment of CHS genes in division I. Red and blue triangles indicate type I and type II specificity-determining sites identified by SPEER-SERVER (Chakraborty et al., 2012), respectively. The letters f and s represent functional sites and structural sites identified by ConSurf (Ashkenazy et al., 2010), respectively.
